# Beaver dams attenuate flow: A multi‐site study

**DOI:** 10.1002/hyp.14017

**Published:** 2021-01-01

**Authors:** Alan Puttock, Hugh A. Graham, Josie Ashe, David J. Luscombe, Richard E. Brazier

**Affiliations:** ^1^ Centre for Resilience in Environment, Water and Waste (CREWW), Geography, College of Life and Environmental Sciences University of Exeter Exeter UK

**Keywords:** beaver, beaver dams, catchment management, flood peaks, flow attenuation, flow regimes, hydrology, natural flood management

## Abstract

Beavers can profoundly alter riparian environments, most conspicuously by creating dams and wetlands. Eurasian beaver (*Castor fiber*) populations are increasing and it has been suggested they could play a role in the provision of multiple ecosystem services, including natural flood management. Research at different scales, in contrasting ecosystems is required to establish to what extent beavers can impact on flood regimes. Therefore, this study determines whether flow regimes and flow responses to storm events were altered following the building of beaver dams and whether a flow attenuation effect could be significantly attributed to beaver activity. Four sites were monitored where beavers have been reintroduced in England. Continuous monitoring of hydrology, before and after beaver impacts, was undertaken on streams where beavers built sequences of dams. Stream orders ranged from 2nd to 4th, in both agricultural and forest‐dominated catchments. Analysis of >1000 storm events, across four sites showed an overall trend of reduced total stormflow, increased peak rainfall to peak flow lag times and reduced peak flows, all suggesting flow attenuation, following beaver impacts. Additionally, reduced high flow to low flow ratios indicated that flow regimes were overall becoming less “flashy” following beaver reintroduction. Statistical analysis, showed the effect of beaver to be statistically significant in reducing peak flows with estimated overall reductions in peak flows from −0.359 to −0.065 m^3^ s^−1^ across sites. Analysis showed spatial and temporal variability in the hydrological response to beaver between sites, depending on the level of impact and seasonality. Critically, the effect of beavers in reducing peak flows persists for the largest storms monitored, showing that even in wet conditions, beaver dams can attenuate average flood flows by up to ca. 60%. This research indicates that beavers could play a role in delivering natural flood management.

## Graphical abstract

Four sites were monitored where beavers have been reintroduced in Great Britain to understand the impacts of beaver dams upon flow attenuation.
Analysis of >1000 storm events showed an overall trend of reduced total stormflow, increased peak rainfall to peak flow lag times and reduced peak flows.
Analysis showed flow attenuation impacts due to beaver damming to persist during largest flood events, whilst additionally flow regimes became overall less flashy following beaver impact.

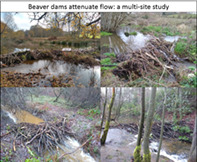

## 1 INTRODUCTION

Beavers have the capacity to modify freshwater ecosystems extensively (McKinstry et al., 2001), creating diverse wetland habitats with significant biodiversity benefits (Brazier, Elliott, et al., 2020; Law et al., 2016; Rosell et al., 2005; Willby et al., 2018). Beavers are considered a keystone species due to their engineering, notably the construction of dams and impoundment of large volumes of water (Hood & Bayley, 2008). Such alterations to ecosystem structure impact upon hydrological functioning by increasing water storage (Grygoruk & Nowak, 2014; Westbrook et al., 2020) but also a change in downstream connectivity (Macfarlane et al., 2015). The impact upon hydrological functioning can be summarized as an increase in lateral connectivity, with dams pushing water out sideways onto floodplains (Puttock et al., 2017). Such a change has been shown to result in flow attenuation characterized by increased water retention and increased rainfall to peak flow lag times (Burchsted & Daniels, 2014; Green & Westbrook, 2009; Westbrook et al., 2020) and reduced flows (Beedle, 1991; Burchsted & Daniels, 2014) downstream of beaver sites. These impacts result due to increased water storage and increased structural roughness created by dams reducing downstream connectivity during storm flow events (Puttock et al., 2017). Conversely, water storage in ponds and overall flow regime attenuation can also result in a persistence of downstream hydrological connectivity during low flow or drought periods via the slowed release of water and maintenance of base flows (Fairfax & Small, 2018; Pilliod et al., 2017).

Flooding is an economically and socially costly natural hazard, predicted to increase under future climate scenarios (Dadson et al., 2017). There is also a growing recognition of the multiple benefits of working with natural processes to deliver ecosystem services with societal benefits including flood risk reduction (Lane, 2017). Natural flood management or hybrid “soft” engineering approaches may provide holistic, catchment‐based flood management options (Hewett et al., 2020; Lane, 2017; Wilkinson et al., 2014), increasing the resilience or effectiveness of existing conventional “hard” engineering defences and delivering wider environmental and societal benefits (Lane, 2017). They may also provide alternatives at the local scale where hard engineering is not viable or affordable (Short et al., 2019). Beavers have been posited as a possible natural flood management option (Environment Agency, 2014). However, with a few exceptions (e.g., de Visscher et al., 2014; Nyssen et al., 2011) the existing hydrological research into the impacts of beaver has been undertaken in North America in extensively managed landscapes (Burns & McDonnell, 1998; Green & Westbrook, 2009). Previous work on a small, first‐order stream in England demonstrated the ability of beavers to transform a single channel into a series of ponds (Puttock et al., 2015), store large volumes of water, attenuate flow regimes leading to reduced peak and total flows downstream during storm events (Puttock et al., 2017) and also trap sediment and nutrients (Puttock et al., 2018). On a 2nd order stream in a forest mountain catchment in Belgium, beaver dams were shown to result in flow attenuation by reduced flood peaks and increased low flows (Nyssen et al., 2011). Modelling on Bavarian river systems (Neumayer et al., 2020) showed alternation to flow regimes and flow attenuation. Whilst these studies illustrate the potential of beaver dams to attenuate flooding, there is little empirical understanding into the impact of beaver upon hydrological functioning across the range of scales where damming may occur (Graham et al., 2020) in intensively managed landscapes representative of large areas of northern Europe.

Most European catchments have become a product of human activity with associated problems including hydrological extremes, diffuse pollution and soil erosion (Hewett et al., 2020). In such landscapes it has been suggested that beaver previously exerted a large influence on riverine structure and function (Brown et al., 2018). Hunted to near extinction, the Eurasian beaver (*Castor fiber*) has now been reintroduced to much of its former range (Halley et al., 2012), with recent reviews estimating populations at 1.5 million (Halley et al., 2020). In Great Britain (GB), where beavers were extirpated and thus absent by the 16th Century (Conroy & Kitchener, 1996), there are now an increasing number of controlled release sites and expanding wild populations (Brazier, Puttock, et al., 2020; Campbell‐Palmer et al., 2018). Such population increases add urgency to the need for increased understanding of beaver impacts to inform catchment management strategies, to maximize opportunities but also mitigate conflict (Auster et al., 2019, 2020; Campbell‐Palmer et al., 2016). Key examples of conflicts recorded GB landscapes include agricultural crop feeding, burrowing and damming that puts agriculture or critical infrastructure at risk (Campbell‐Palmer et al., 2020).

Working across spatial scales represented by differences in drainage density at the small catchment size (with second to fourth order channels) and catchments dominated by both lowland agriculture and forestry, this study applied a standardized suite of hydrological analyses to address the following hypotheses:

H1. Hydrological event peak flows and flashiness are reduced following beaver modification.

H2. Peak flow attenuation can be attributed beaver engineering, particularly the construction of dams.

## 2 METHODS

### 2.1 Study sites

**FIGURE 1:**
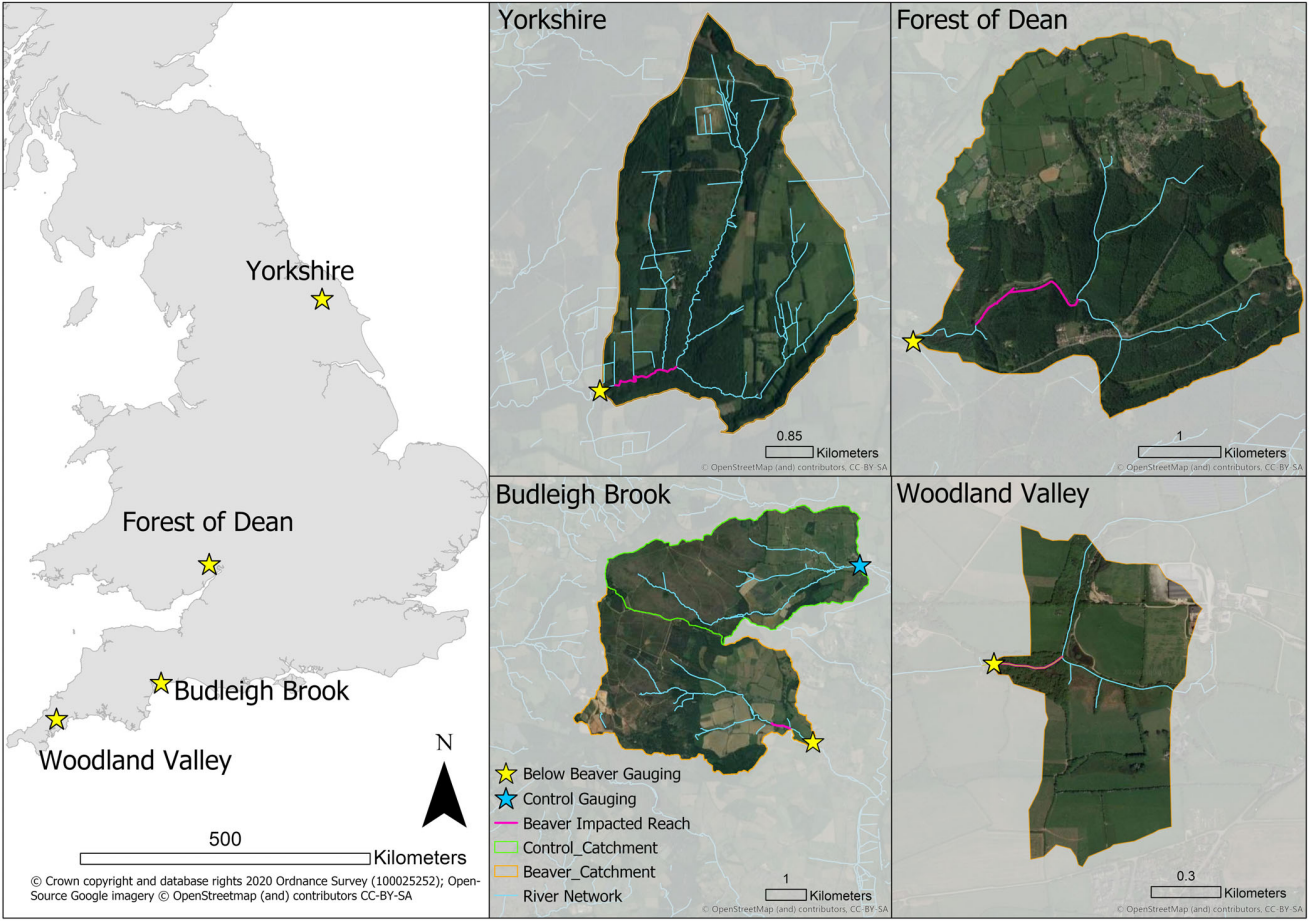
Left: Study site locations within England. Right: Catchment areas for the four study sites indicating the location of beaver complexes and flow gauging

Hydrological monitoring was undertaken across four sites (Locations in Figure 1 with additional aerial imagery of sites in SI.6) in England adopting a multi‐site before‐after beaver experimental design, that is, monitoring downstream of beaver reintroduction sites was undertaken prior to release, then continued post‐release to understand impacts upon hydrological functioning, relative to rainfall. At one site (Budleigh Brook) where beavers established a territory, a suitable control site was fortunately available allowing for a full Before‐After‐Control‐Impact (BACI) experimental design (Bilotta et al., 2016). Two of the beaver impacted sites (Woodland Valley and Budleigh Brook) had agriculturally dominated catchments (both intensive and extensive grassland and some arable), whilst the other two beaver impacted sites (Forest of Dean and Yorkshire) were forestry dominated. Beaver dam modelling presented in Graham et al. (2020) showed all sites to have high capacities for supporting dam sequences, indicating that they were suitably representative of where beaver dam sequences may be expected. The authors were not responsible for the release of beavers, the timing and location of releases or, in the case of Budleigh Brook, natural colonization could not be prescribed. Therefore, the duration of monitoring and therefore the number of hydrological events analysed varies between sites and before/after beaver colonization. We have therefore adopted statistical approaches that can accommodate such an experimental design but acknowledge that the power of derived statistical models will vary between sites as a consequence.

#### 2.1.1 Woodland valley

Woodland Valley (WV) hosts the Cornwall beaver project and is situated on a 2nd order stream. The site experiences a temperate climate with an annual mean maximum temperature of 13.5°C and mean annual rainfall of 1017.4 mm (Met Office, 2020). In June 2017, a pair of beavers were introduced to a 1.5 ha enclosure, dominated by willow and birch woodland, in addition to gorse scrub. The site has a 134 ha contributing area dominated by grazed grassland (~70%) and some arable that didn't change through the monitoring period. Beavers created 7+ dams in addition to damming and raising the water level in a pre‐existing pond. Further information on the project and partnership involved can be found at: https://www.cornwallwildlifetrust.org.uk/what-we-do/our-conservation-work/on-land/cornwall-beaver-project.

Flow in and out of the site was monitored to create a continuous record of discharge from November 2015 to March 2019. A smooth lined culvert on the channel leaving the enclosed site was instrumented with an in‐situ submersible pressure transducer (IMSL–GO100, Impress, United Kingdom) situated in a stilling well. Water level was recorded on a 15 min time step. Water level was converted to discharge using Manning's equation with a surface roughness value of 0.015 =:

$$Q=\frac{\mathrm{KA}R^{0.667}S^{0.5}}{n}$$

Q = flow rate; A = cross sectional area of flow; R = hydraulic radius (cross sectional area divided by wetted perimeter); S = slope of channel (rise) n = Manning's surface roughness value. K = constant (1 for metric measurements).

#### 2.1.2 Budleigh Brook and control site

A free‐living beaver group established themselves on the 3rd order Budleigh Brook in the River Otter catchment, Devon. The population has been present since January 2017. The occupied section of channel is ~1 km long and has contained up to 6 dams. It has a 6.3 km^2^ contributing catchment area of mixed landuse, (intensively managed grassland, pig farming, arable, heath and woodland). The site experiences a temperate climate with an annual mean maximum temperature of 12.6°C and mean annual rainfall of 1065.3 mm (Met Office, 2020). Further information about the trial can be found at: https://www.devonwildlifetrust.org/what-we-do/our-projects/river-otter-beaver-trial.

An Environment Agency (EA) gauging station is located 700 m downstream of the lowest beaver dam. No substantial hydrological inputs occur between the site and the gauge. The gauge measures water depth, at 15 min intervals, within a stilling pond, upstream of a trapezoidal weir with low flow trapezoidal notch. The weir was rated with an area‐velocity flow meter (NivuFlow Mobile 750, Nivus, Germany) for two months. A flow rating equation (data repository in Data S1) was generated between flow and depth using piecewise spline regression as described in Fenton (2018); undertaken using the splines package (R core team, 2020). The period of monitoring extends from July 2009 – March 2020.

The neighbouring catchment is Colaton Brook a 3rd order stream with a contributing catchment area of 5.5 km^2^. The landuse is mixed, comprising heathland, managed grassland, arable, and woodland. No beavers have been observed in the catchment. A downstream EA gauging station provides 15 min interval flow measurements. The comparable size and locale of this catchment makes it a highly suitable control catchment, which can be used to evaluate the effect of beavers on Budleigh Brook in a BACI framework.

#### 2.1.3 Forest of Dean

The Forest of Dean beaver project is situated on the 3rd order Greathough Brook, Gloucestershire. The site experiences an annual mean maximum temperature of 14.4°C and mean annual rainfall of 733.5 mm (Met Office, 2020). In July 2018, a pair of beavers was introduced to a 6 ha enclosure, dominated by mixed broad‐leaf woodland. The site has a 410 ha contributing area dominated by mixed broad leaf woodland and some roads/ urban areas. Since release, beaver have created 3 dams. Further information on the project can be found at: https://www.forestryengland.uk/beavers-greathough-brook-forest-dean.

The site was from October 2017 to May 2019 at which point beavers were temporarily removed from the site for a project pause (the monitoring time series used included 9 months of pre‐beaver baseline data and 10 months of post‐beaver data). A monitoring station on a culvert leaving the site was instrumented with an in‐situ submersible pressure transducer (MX2001, HOBO ONSET, USA) recording on a 15 min time step. Water level through the culvert was converted to discharge using Manning's equation using a roughness coefficient of 0.015 for a smooth lined culvert [Equation (2)].

#### 2.1.4 Yorkshire

On 17 April 2019, a beaver pair were released into a 16 ha enclosure in Cropton Forest, North Yorkshire, on a 4th order stream (Sutherland Beck) as part of a five‐year scientific trial. The site has a 747 ha catchment upstream and the landuse is a mixture of widely spaced beech and pine with a rhododendron understorey, plantations of Norway Spruce, Scots Pine, Douglas fir and stands of Silver Birch (Forestry England, 2020). The site experiences an annual mean maximum temperature of 11°C and mean annual rainfall of 978.9 mm (Met Office, 2020). The site was part of a project focusing on natural measures to alleviate flooding downstream. Information on the Slowing the Flow project in the River Seven and Pickering Beck catchments, can be found at: https://www.forestresearch.gov.uk/research/slowing‐the‐flow‐at‐pickering/slowing‐the‐flow‐at‐pickering‐about‐the‐project/. As part of this initiative multiple timber bunds are in place across the channel. However, they have not changed post‐beaver reintroduction and, during the analysis, there was no interaction recorded between beavers and these structures so they were treated as a constant and not explicitly considered in analysis. Further information on the project can be found at: https://www.forestryengland.uk/beaver-trial-cropton-forest.

The site was monitored to create a continuous record of discharge from December 2018 to March 2020 (this monitoring included 5 months of pre‐beaver baseline data and 11 months of post‐beaver data). A monitoring station on the channel leaving the site was instrumented with an area‐velocity flow meter (NivuFlow Mobile 750, Nivus, Germany) and an in‐situ pressure transducer (MX2001, HOBO ONSET, USA), recording on a 15 min time step. Discharge from the area‐velocity flow meter was checked against level data from the pressure sensor.

### 2.2 Data analysis

Links to full data analysis repositories are included in Data S1.

#### 2.2.1 Rainfall data collection

Whilst sites were equipped with a tipping bucket rain gauge (RG3M, HOBO ONSET, USA), rainfall is spatially variable and data from a single rain gauge can be non‐representative, particularly in forested catchments (Younger et al., 2009; Zeng et al., 2018). Therefore, rainfall radar data, derived from the NIMROD system (Met Office, 2003), was used across sites. NIMROD data are provided as gridded total rainfall with resolutions of 1 km and 5 min, respectively. Total rainfall for each time step was extracted for each site's contributing catchment area and converted to mean rainfall rate, before aggregating to 15 min to align with the temporal resolution of flow data. Data download and conversion (Data S1) was conducted using Python 3 and raster statistics were extracted with R using the exactextractr package (Bastion, 2020).

#### 2.2.2 Data preparation and storm event extraction

**FIGURE 2:**
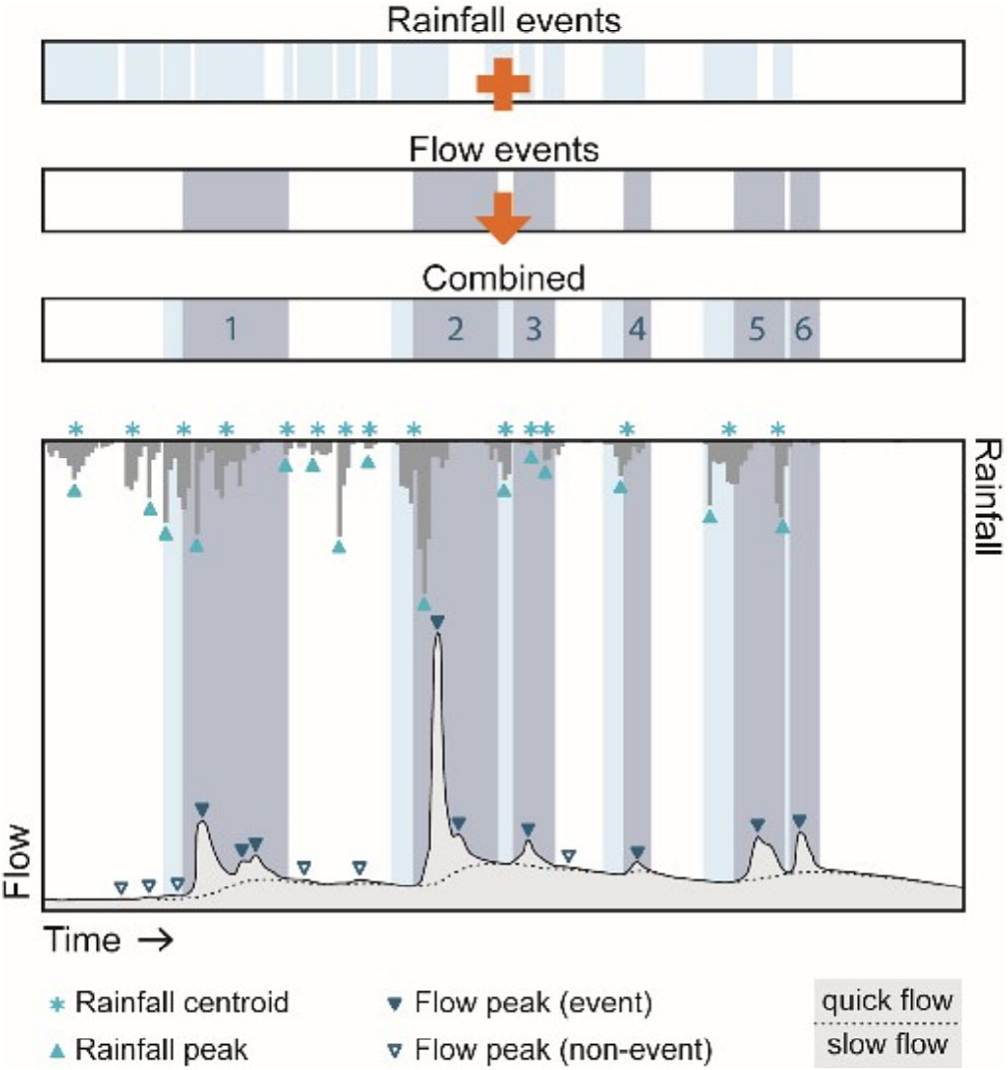
A conceptual figure depicting the event extraction methodology. Periods of continuous rainfall are identified alongside corresponding flow events where quick flow exceeds slow flow. The durations of both rainfall and elevated flow are combined to create an event window which is used to extract hydrological information for a given storm event

The systematic extraction of rainfall‐runoff events and corresponding metrics was undertaken using a semi‐automated rules‐based approach for the identification and pairing of rainfall and flow geometries from sub‐hourly observations (Ashe et al., 2019; Deasy et al., 2009; Glendell et al., 2014; Ladson et al., 2013; Luscombe, 2014; Puttock et al., 2017) summarized in Figure 2. Data were sub‐sampled at 15 min intervals and pre‐processed for quality control (Ashe et al., 2019). The automated systematic approach for flow event extraction is sensitive to low flow variability in the discharge time series. Therefore, we used an automated cleaning strategy. This approach calculates rolling quantiles for a specified time window (12.5 h) at the 25th and 75th percentile, (Q25th and Q75th respectively). A rolling quantile for the 70th percentile for a one month period is also calculated (MQ70). Where (Q75th ‐ Q25th) > MQ70, the flow is considered to be elevated and any fluctuation in flow is driven by precipitation; therefore measured Q is used. Where (Q75th ‐ Q25th) < MQ70, the flow is considered to be low and not responding to a flow event; we therefore used a 7.5 h rolling mean for Q in place of measured Q to smooth out sensor noise during low flows. No cleaning was applied to flow event peaks and thus did not alter the observed results derived from the event extraction process. Slow flow (equivalent to base flow) and quick flow (equivalent to stormflow) was estimated by implementing flow separation on the time series after Ladson et al. (2013). Analysis was done in R 3.6.3. (R Core Team, 2020). Event extraction time series for each site are included in data repository with an example in Figure S1. Event metrics were calculated for each event (Data S1). Misidentified events were located through visual inspection and removed from analysis.

#### 2.2.3 Statistical design and analysis

The statistical design used in this study focusses on the before‐after (BA) intervention comparison as used previously in hydrological studies including beaver (Hill & Duval, 2009; Nyssen et al., 2011) and related river or restoration studies (Grayson et al., 2010; Sear et al., 2006). The lack of control monitoring increases uncertainty that another, unmeasured, factor could cause change (Downes et al., 2002). However, to our knowledge there were no major land use changes or known confounding factors during the monitoring period. The monitoring of four different sites further strengthens the robustness of findings where common trends are observed across sites. Additionally, at one site (Budleigh Brook), beavers colonized an area with suitable control monitoring, allowing the opportunistic adoption of full Before‐After‐Control‐Impact (BACI) experimental design as outlined in (Bilotta et al., 2016). BACI analysis was not possible across all sites because no suitable control catchments were available. Whilst selection of controls at the catchment scale is complex, due to the probability of confounding processes (Lane, 2017), it is recognized as a stronger analytical approach (Shuttleworth et al., 2019). Therefore, we adopted a mixed experimental design with a BA design across four sites and a repeated analysis of one of these sites, using a BACI design (as in Bilotta et al., 2016). Should results from the BACI site align with those from the BA sites, greater confidence can be held in the findings from BA sites.

Hydrological data from storm events is non‐normally distributed and as such all statistical analysis was undertaken using appropriate tests; either non‐parametric (as in Table 2) or generalized linear models (GLM). Additionally, the experimental design did not give us control over when beavers were released into or impacted upon sites or when and how many rainstorm events occurred during the monitoring period. As such, an unbalanced dataset, both between sites and between Before‐After periods was inevitable. This imbalance is often an unavoidable issue for field researchers with access to limited, or in this case pre‐determined, field sites (Warton et al., 2016). Rather than exclude data from analysis which risks incurring bias, loss of precision or obscuring key information on system function (Shaw & Mitchell‐Olds, 1993), statistical approaches were carefully selected that could handle unbalanced datasets to support robust conclusions.

Statistical analysis was undertaken in R 3.6.3. (R Core Team, 2020) with data manipulation, summary statistics and plotting undertaken using the tidyverse (Wickham et al., 2019). Q5:Q95 ratio was used as a simple flashiness index (Jordan et al., 2005). The statistical significance for differences between pre‐ and post‐beaver groups for summary statistics were determined using the non‐parametric Mann–Whitney *U* test.

Direct comparison of hydrological metrics pre‐ and post‐beaver, provides an indication of beaver impact. However, this does not consider the amount of rainfall. We therefore used GLMs, with a Gamma error distribution and identity link functions, where event rainfall is the control variable, event peak Q is the response variable and beaver presence is considered as an additive explanatory variable.

The model form is shown below:

*Q peak ~ Total Rainfall + Beaver Presence*.

This allows for testing the effect of beaver on peak flows, relative to contributing event rainfall. GLMs were chosen over linear regression, due to their ability to cope with non‐normally distributed response variables (Dunn & Smyth, 2018). As smaller flow events are more common than large events the error distribution of event peak flows for all sites has a Gamma distribution. Unique regression models were designed for each site, negating issues of sample size imbalance between sites. Analysis was undertaken using the glm2 R package (Marschner, 2011). Critically, this approach can also handle unbalanced sample size (i.e., unequal factor levels) as General Linear Models do not require equal group sizes (Dunn & Smyth 2018; Venables & Ripley, 2002; Warton et al., 2016). Unequal group sizes can have two important effects relevant here: (i) the power of the model is limited by the size of the smallest group (Shaw & Mitchell‐Olds, 1993) and (ii) Care should be taken when selecting a model and interpreting its results from an unbalanced design to ensure the hypothesis may be addressed (Hector et al., 2010; Warton et al., 2016). In addressing the first point; we acknowledge the difference in statistical power across our different sites. Regarding point ii; imbalance is a greater problem with sample sizes smaller than those presented in this paper (Warton et al., 2016) and any issues can be identified during model evaluation with visual diagnostic plots. This was carried out using the “performance” package in R (Lüdecke et al., 2020).

Large storms are of most interest for catchment management and flood risk (Puttock et al., 2017). As with most empirical hydrological monitoring projects, the long‐term time series data required to calculate robust storm recurrence intervals was not available and there are significant limitations in trying to predict return periods from limited time series (Pomeroy et al., 2016). Therefore, exceedance limits were used to evaluate the effect of beaver on peak flows. At each site, a subset of events was created where peak flows exceeded the Q5 flow exceedance value. The GLM analysis was repeated for this high‐flow subset to test if there was significant difference between pre‐ and post‐beaver periods for events where flow percentage exceedance values were greater than 95th percentile. Q5 was chosen as a recognized high flow metric (Jordan et al., 2005; Kamamia et al., 2019).

To investigate how impact varied over different hydrological seasons, GLMs were produced for the full dataset across all sites including hydrological year as an interactive covariate which, in Great Britain, is widely recognized as starting on the 1st of October (NRFA, 2018). It has been shown that heaviest rain events typically occur in the winter or wet half of the hydrological year between October and March and this is a key driver over extreme flood events (Lavers et al., 2011). In line with previous research (Lavers et al., 2011, 2013; Puttock et al., 2017) the “wet season” was defined as the period between 1st October and 1st April with the other half of the year defined as the “dry season”. The model form is described below.

Q peak ~ Total Rainfall + Beaver Presence * Hydrological Season.

For the Budleigh Brook site, a suitable control site was monitored in the neighbouring Colaton Brook catchment (Figure 1). Therefore the GLM analysis described above was repeated to investigate the impact of a beaver dam complex on: (i) all measured peak flows, (ii) peak flows >Q5 exceedance levels and (iii) with hydrological season as a covariate. However, for all of these GLMs, the models were also run with the control site data included, with site used as an additional interactive covariate, in line with BACI sampling designs (Bilotta et al., 2016). The formulations of the models are:

Q peak ~ Total Rainfall + Beaver Presence * Site.

Q peak ~ Total Rainfall + Beaver Presence * Hydrological Season * Site.

The inclusion of a control site allows for a greater degree of confidence in the observed response reported in the GLMs. If a significant difference in flows can be attributed to beaver, then this will be reflected in the interaction between site and beaver presence.

Estimated marginal means (i.e., adjusted or least‐squares means), along with associated standard errors, were calculated using the emmeans R package (Length, 2020) for all GLMs to compare differences in mean peak flows before and after beaver, over different hydrological seasons and, where the control is used, between control and impacted sites. Estimated marginal means (emmeans) are useful for interpreting the outputs of regression analysis where the difference between, and or the effect of, factor levels is of interest (Castorani et al., 2018; Piepho & Edmondson, 2018). Furthermore, emmeans are designed to handle factor levels of different sizes by adding equal weight to each cell (or group). This eases the interpretation of model predictions in this unbalanced case (Length, 2020).

## 3 RESULTS

### 3.1 Hydrological response across four sites before and after beaver

Summary results from all events before and after beaver are illustrated in Table 2. In total across the four sites, 1153 events were extracted with 582 occurring before beaver and 571 following beaver reintroduction. Across all sites, there was a trend of total stormflow and the peak flow reduction following beaver impact (Figure S2). Results from Mann–Whitney *U* tests (Table 2) show before after differences to be significant (*p* < 0.05) at all sites apart from Yorkshire which, in addition to having the shortest monitoring period, was the only site to have a significant difference in event rainfall (Figure S3), with storms having greater median rainfall (*p* < 0.05) in the post beaver period. The time between peak rainfall and peak discharge in a storm event (lag time) was shown to increase across all sites apart from Yorkshire, with the increase being significant (*p* < 0.05) at Woodland Valley and Budleigh Brook.

Additionally, Q5:Q95 ratios were calculated as a flashiness index from the whole time series across the sites, before and after beaver impact. All sites showed a reduction in Q5:Q95 after beaver impact (Table 2). This indicates that overall flow regimes were less “flashy” or more attenuated with less difference between high (Q5) and low (Q95) flow periods when beaver were present. In addressing hypothesis 1, results indicate that across the four sites there had been a change in flow regimes following beaver reintroduction. Although it must be recognized that summary statistics presented in Table 2 do not in isolation prove a causal link between change and beaver engineering as they do not account for variability in rainfall. Therefore, the following sections address hypothesis 2, to understand whether changes to observed peak flows can be attributed to beaver activity.

**FIGURE 3:**
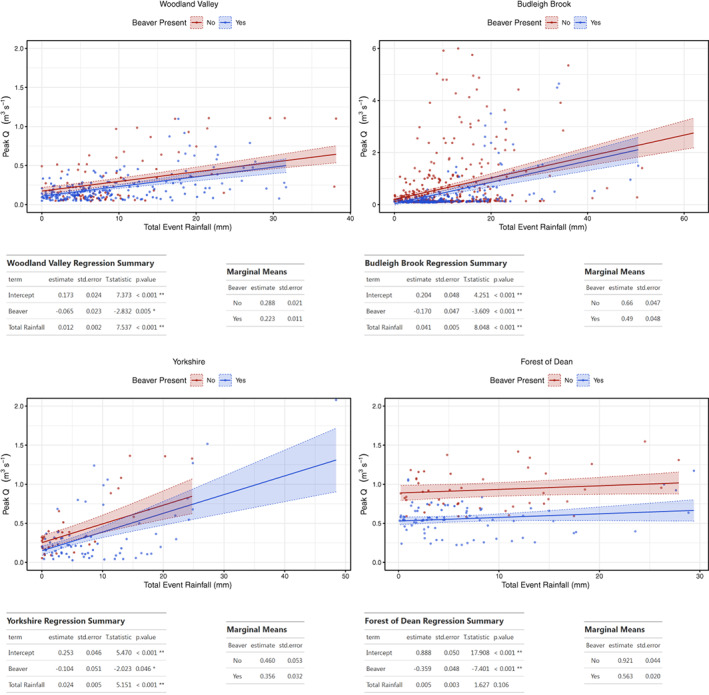
GLM model results between peak Q and total event rainfall, before and after beaver impact across all sites for all recorded storm events. Top: model output plots; Bottom: model summary and marginal mean values for each site

GLM analysis was undertaken for all event data across the sites with beaver presence/absence as an additive variable (results and summary test statistics in Figure 3). As shown by marginal means, peak flow showed a reduced response to rainfall across all sites. In regression summary tables for each site, the estimate value gives the modelled magnitude of change in peak flow (m^3^ s^−1^), and also the direction (increase or reduction) for every unit of total event rainfall (mm). Models showed beaver impact to result in a statistically significant (*p* < 0.05) reduction in peak flow. The estimate value for these reductions range from −0.359 m^3^ s^−1^ at the Forest of Dean to −0.065 m^3^ s^−1^ at Woodland Valley.

**FIGURE 4:**
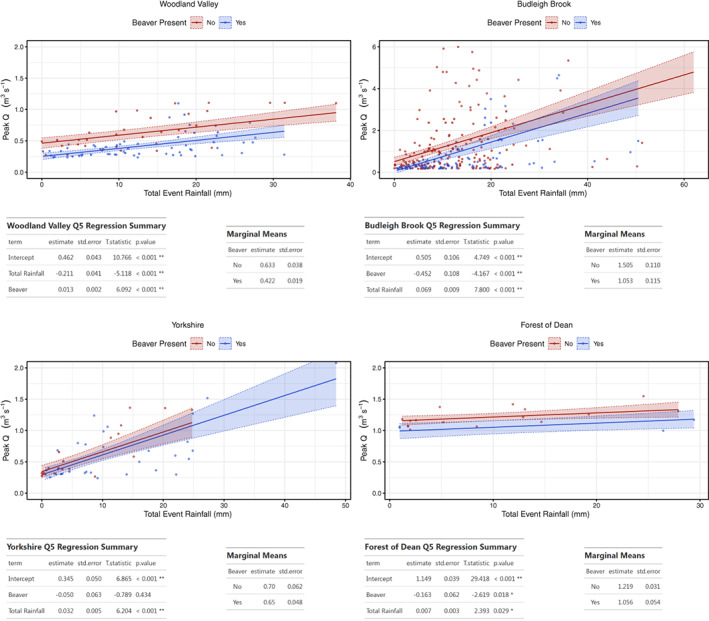
GLM model results between peak Q and total event rainfall, before and after beaver impact across all sites for events larger than the Q5 exceedance level. Top: model output plots; Bottom: model summary and marginal mean values for each site

To investigate whether flow attenuation persisted for large events, identical analysis was undertaken on a > Q5 subset of events (Figure 4). Emmeans and estimates showed even for this subset of the largest events monitored, there was still a reduction in peak flow across all sites. Notably, estimated reduction effects of beaver upon peak flow per unit rainfall during large events increased at the two more established beaver impacted sites with agriculturally dominated catchments (Woodland Valley −0.065 to −0.211 m^3^ s^−1^ and Budleigh Brook −0.170 to −0.452 m^3^ s^−1^), but reduced at the less established sites with fewer dams and woodland dominated catchments (Yorkshire −0.104 to −0.050 m^3^ s^−1^ and Forest of Dean −0.359 to −0.153 m^3^ s^−1^).

**FIGURE 5:**
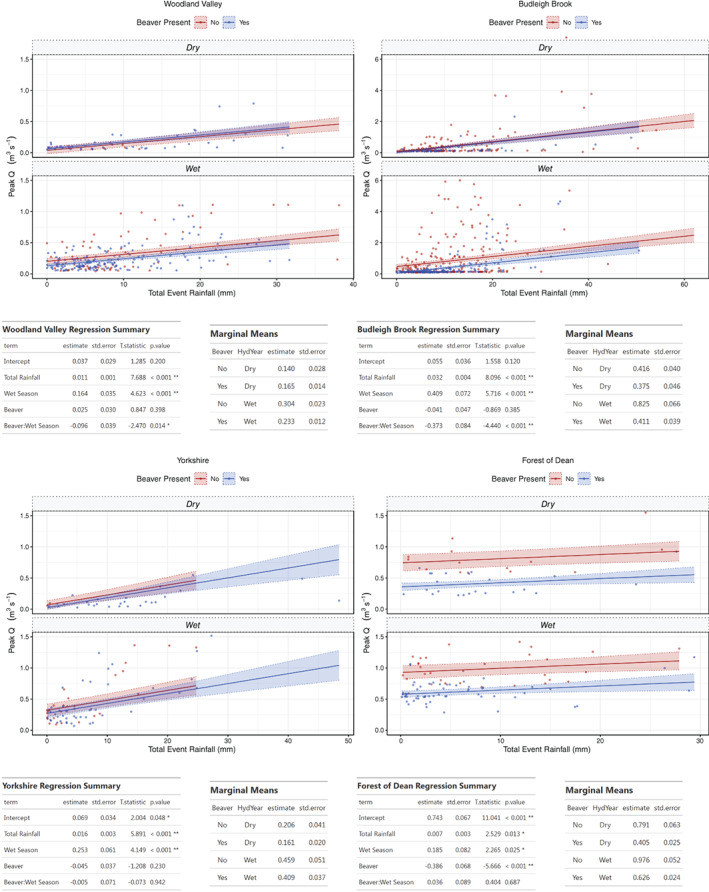
GLM model results between peak Q and total event rainfall, before and after beaver impact across all sites with the addition of season as an effect. Top: model output plots; Bottom: model summary and marginal mean values for each site

To determine if seasonality affected the impact of beaver upon peak flows, hydrological year was included as an interactive covariate in GLM analysis (Figure 5). Model summary statistics (Figure 5) show that, for all sites, season has a significant effect (*p* < 0.05), with an increased peak flow response to rainfall during the wet season. Results across all sites apart from the Forest of Dean also show the interactive effect between the presence of beaver and wet season to be negative (i.e., beaver activity leads to a reduction in peak flow) and that the impact of beaver presence upon peak flow is greatest during the wet season of the year. This effect is statistically significant at both Woodland Valley and Budleigh Brook, sites with agriculturally dominated catchments and where most events were monitored (908 total).

Observed differences in impact of beaver upon the response of peak flow to rainfall, between seasons, is further illustrated in the tables of emmeans from model outputs (Figure 5). Across all sites, emmeans estimates from the models are higher during the wet season again illustrating that, during the wet half of the year, a greater peak flow response to rainfall will be predicted. Emmeans values show a general trend of reduced peak flow values after beaver reintroduction. However, as illustrated most clearly for Woodland Valley and Budleigh Brook, this reduction in peak flow impact of beaver is greater during the wet season. For example, at Woodland Valley, after beaver reintroduction there was actually a small (0.025 m^3^ s^−1^) increase during the dry season, but a reduction of 0.071 m^3^ s^−1^ (23%) during the wet season. At Budleigh Brook there was a reduction of 0.041 m^3^ s^−1^ (10%) during the dry season and a reduction of 0.414 m^3^ s^−1^ (50%) during the wet season. At the two forested sites there was less of a clear seasonal differentiation with Yorkshire showing a 22% reduction during the dry season and an 11% reduction during the wet season and Forest of Dean showing a 48% reduction after beaver during the dry season and a 36% reduction during the wet season.

### 3.2 Hydrological response at a site before and after beaver compared to a control site

**FIGURE 6:**
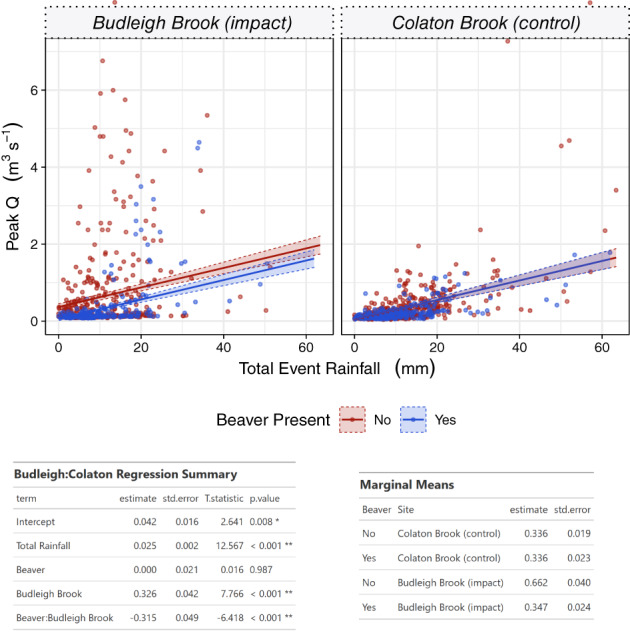
GLM model results between peak Q and total event rainfall, before and after beaver impact at Budleigh Brook and compared to a control site (Colaton Brook). Top: model output plots; Bottom: model summary and marginal mean values for each site

To investigate hypothesis 2 further, at Budleigh Brook a suitable control site, with a comparable data record (634 events over the same time period), was available. Therefore, adopting a full BACI approach, GLM analysis was run, incorporating site as an interactive effect with results illustrated in Figure 6. BACI results for Budleigh Brook add further weight to support the acceptance of Hypothesis 2 with the combined effect of site and beaver presence shown to result in a significant reduction in peak flows (*p* < 0.01). This effect is most clearly shown in the marginal means; at the control site (Colaton Brook), the modelled effect of rainfall was 0.33 m^3^ s^−1^ both before and after the period where beaver colonized Budleigh Brook. In contrast, at Budleigh Brook there was a reduction in mean peak flow from 0.66 to 0.35 m^3^ s^−1^ (47%) after beaver reintroduction.

Identical analysis was undertaken on a data subset with flows greater than Q5 (Figure S4). Results showed the attenuation effect of beavers, at the occupied Budleigh Brook site, persisted for large events with a significant reduction in peak flows (*p* < 0.01) in contrast to the control. Marginal mean values from GLM analysis (Figure S4) show a mean peak flow of 0.50 m^3^ s^−1^ before and 0.48 m^3^ s^−1^ after at the control site. In contrast Budleigh Brook, the beaver impacted site, showed a reduction from 1.53 to 0.65 m^3^ s^−1^ for Q5 events after beaver were reintroduced (57% reduction).

**FIGURE 7:**
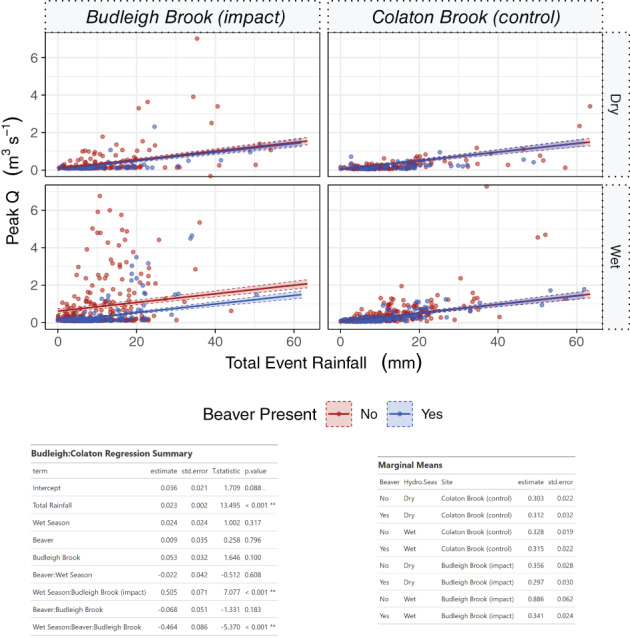
GLM model results between peak Q and total event rainfall, before and after beaver impact at Budleigh Brook and compared to a control site (Colaton Brook) with the addition of season as a fixed effect. Top: model output plots; Bottom: model summary and marginal mean values for each site

Analysis was also undertaken for Budleigh Brook incorporating both season and control data (Figure 7). Results showed the combined effect of beaver presence, site and season to be statistically significant. Marginal means allow further interpretation of this multi‐parameter analysis (Figure 7), effectively showing no change at the control throughout seasons and the period of beaver impact (all have an effect of ca 0.3 m^3^ s^−1^). In contrast the beaver impacted site showed a reduction from 0.36 to 0.30 m^3^ s^−1^ (17%) after beaver reintroduction in the dry season, but a greater reduction from 0.87 to 0.34 m^3^ s^−1^ (62%) during the wet season.

## 4 DISCUSSION

### 4.1 Alteration of flow regimes by beaver dams

Analysis of storm events, across four sites, demonstrated that flow regimes were altered after the construction of beaver dam complexes, with an overall trend of reduced peak flows, reduced total stormflow, and increased lag times. Additionally, the overall “flashiness” of flow regimes was reduced. Results support the acceptance of Hypothesis 1 that there was a change in flow regime and hydrological response to storm events following beaver modification. Furthermore, before‐after analysis across four sites and full BACI analysis at one site significantly attributes changes in peak flows to beaver impact, supporting the acceptance of Hypothesis 2.

Results support previous research showing beaver impact can alter flood hydrographs, reduce the peak discharge of floods and increase lag times (Burns & McDonnell, 1998; Green & Westbrook, 2009; Nyssen et al., 2011; Puttock et al., 2017). The attribution of flow attenuation to beaver supports research highlighting the need to acknowledge the influence of biotic factors upon river form and process (Johnson et al., 2019). More specifically, multiple previous studies have identified beaver modified landscapes, as a potential cause of flow attenuation (Green & Westbrook, 2009; Gurnell, 1998; Pollock et al., 2007). When presenting the reductions in peak flows and total stormflows it is important to understand that water is not disappearing, but is instead being released downstream more slowly. The attenuation impact of beavers has been ascribed primarily to increased water storage in beaver pond sequences (Westbrook et al., 2020). That is, at the Budleigh site >1000 m^2^ of ponded area was created (Brazier, Elliott, et al., 2020) whilst recent estimates at the Woodland Valley site indicate >2000 m^2^. A previous study at a smaller site (Puttock et al., 2017) showed >1000 m^2^ of ponded area to result in over a million litres of water storage in 13 beaver ponds. Attenuation is also attributed to increased hydrological roughness from dams and surrounding floodplain wetlands (Puttock et al., 2017), increasing lateral connectivity (Macfarlane et al., 2015), diverting water sideways into ponds, soil and also ground water (Feiner & Lowry, 2015; Westbrook et al., 2006). Increased water storage also lengthens water retention times (Grygoruk & Nowak, 2014; Gurnell, 1998; Woo & Waddington, 1990), leading to slower downstream release; for example, Green and Westbrook (2009), showed the removal of a beaver dam sequence can lead to substantial (81%) increases in flow velocity. The increased surface area of water could also lead to greater evapotranspiration. Though evaporative fluxes were not measured in this study, previous research (albeit in a dryland environment as opposed to the temperate sites herein) has shown evapotranspiration to be 50–150% higher in riparian areas with beaver damming (Fairfax & Small, 2018).

Whilst there is a body of research attributing flow attenuation to beaver activity, this is the first empirical research to analyse hundreds of events, before and after beaver reintroduction, across multiple sites, using a standardized approach. The study thus adds considerable weight to previous research which demonstrates flow attenuation at small or individual sites (Law et al., 2016; Nyssen et al., 2011; Puttock et al., 2017), individual large storm events (Westbrook et al., 2020) or modelled simulations (Neumayer et al., 2020) and quantifies the peak flow and flashiness changes that beaver impacts can deliver across different stream orders and land uses.

This study focuses on high flow periods, but it is worth noting that reduced flashiness observed supports research indicating the slowed release of water from leaky dams may maintain or elevate stream baseflows (Nyssen et al., 2011) during dry periods (Majerova et al., 2015; Puttock et al., 2017; Woo & Waddington, 1990). There is a need for further research into baseflow maintenance, with an increase in hydrological extremes predicted globally (Dadson et al., 2017; Larsen et al., 2009; Romanowicz et al., 2016) both attenuating stormflow *and* maintaining flow and wetness during drought periods (Fairfax & Small, 2018; Gibson et al., 2015) or even fire episodes (Fairfax & Whittle, 2020) which could have major ecological and societal benefits.

### 4.2 Spatial and temporal variation

The overall finding of this study is that beaver impacts result in flow attenuation. However, it is also important to acknowledge that results show variation spatially across sites and temporally, both seasonally and between events.

#### 4.2.1 Variability between sites

Beaver engineering is highly site specific and depends on the existing habitat, building material availability and channel characteristics (Collen & Gibson, 2000; Graham et al., 2020; Woo & Waddington, 1990). It is difficult to define a “typical” dam, although Woo and Waddington (1990) identified some of the multiple ways in which dam structure can influence flow pathways, that is, stream flow can overtop or funnel through gaps in the dams, leak from the bottom of the dams or seep through the entire structure. The impact upon flow velocity will consequently differ (Hering et al., 2001; Woo & Waddington, 1990). It is also important to note the number of dams and density could influence hydrological function. Existing work has discussed the importance of the number of dams in a reach, with beaver dams having the greatest impact on hydrology when they occur in a series (Beedle, 1991; Gurnell, 1998; Nyssen et al., 2011). Ponds located in series provide greater storage and greater roughness (Puttock et al., 2018), resulting in a greater reduction in flow velocities (Green & Westbrook, 2009). Pond sequences have also been shown to reduce the peak flows of 2‐year return floods by 14% whereas individual dams reduced flood peaks of similar events by 5.3% (Beedle, 1991).

Whilst not examined herein in detail, beaver dam numbers and the level of site impact varied throughout the monitoring period and between sites (Table 1). At Woodland Valley (max observed dams = 7) and Budleigh Brook (max observed dams = 6), the longer data record available covered the transformation of each site into a complex beaver engineered wetland, with extensive damming pushing water sideways, connecting the channel and riparian zone. For example, at Budleigh Brook the largest dam extended 60 m across the floodplain (Brazier, Elliott, et al., 2020; Brazier, Puttock, et al., 2020). In contrast, monitoring at the two forested sites, covered the initial period of beaver engineering following release. For example, dams at the Forest of Dean (max observed dams = 3) were still contained within the channel, holding back water in the incised channel to a bankfull height, but not yet pushing water sideways onto the floodplain. Such differences in level of site impact can be seen in the aerial images (Figure S5) and ground images contrasting the impacts of dams during the monitoring period at Woodland Valley and the Forest of Dean (Figure S6). Such differences may explain some of the variation in results observed, that is, the greater reduction in peak flows at the more established sites, with a higher number of dams during large events and the wet season. Whilst research has illustrated that dam sequences have a higher impact than individual dams (Beedle, 1991; Green & Westbrook, 2009), recent research has also show that the configuration of beaver dam analogues also exerts an influence (Munir & Westbrook, 2020) something that must be considered for actual beaver dams too.

**TABLE 1:** Beaver impacted study site characteristics

	Site and catchment characteristics						Monitoring period		
Site	Site size (ha)	Catchment size (ha)	Stream order	Mean annual rainfall (mm)	Dominant landuse	Dam numbers^a^	Beaver impact	Pre beaver (months)	Post beaver (months)
Woodland Valley	2	134	2	1017	Agricultural grassland	1–7	June 2017	19	21
Budleigh Brook	3	630	3	1065	Agricultural grassland	1–6	January 2017	84	38
Forest of Dean	6	410	3	734	Mixed Woodland	1–3	July 2018	9	10
Yorkshire	16	747	4	979	Mixed Woodland	1–3	April 2019	5	11

*Note*: Mean annual rainfall (Met Office, 2015).

^a^ Dam number is given as a range, as this has varied throughout the monitoring period and is highly dynamic. Beaver impact – denotes the point at which beavers began to engineer the sites. Pre‐ and post‐monitoring periods denote in months the length of the time series used for event separation and subsequent analysis.

**TABLE 2:** Summary statistics for all events across all beaver impacted sites

All data	Beaver	Woodland valley	Budleigh Brook	Yorkshire	Forest of Dean
**Event n**	No	78	418	29	57
	Yes	205	207	73	86
**ER ‐ median (IQR)**	No	7.62 (7.47)	8.84 (9.79)	2.74 (8.32)	5.96 (10.53)
	Yes	6.65 (9.07)	8.37 (9.69)	7.17 (10.33)	4.32 (6.33)
	*p value*	*0.902*	*0.951*	0.004*	*0.301*
**Total stormflow Q ‐ Median (IQR)**	No	5839 (19097)	12 099 (11789)	12 782 (50341)	58 344 (49968)
	Yes	5997 (7963)	9745 (6401)	16 254 (23597)	32 699 (25844)
	*p value*	0.012*	0.000*	*0.607*	0.000*
**Peak Q ‐ median (IQR)**	No	0.15 (0.42)	0.15 (0.54)	0.32()	0.89 (0.38)
	Yes	0.15 (0.18)	0.13 (0.13)	0.21()	0.55 (0.22)
	*p value*	0.027*	0.000*	*0.063*	0.000*
**Lag peak to peak ‐ median (IQR)**	No	1.75 (3.25)	2.5 (2.75)	7.5 (13.50)	6.5 (8.25)
	Yes	2.75 (4.63)	3.75 (2.75)	5.6 (4.81)	8.4 (13.19)
	*p value*	0.005*	0.000*	*0.318*	*0.230*
**Q5:Q95 ratio**	No	11.15	2.73	42.37	4.97
	Yes	6.73	2.04	35.72	3.93

*Note*: Event n, total number of events extracted from the time series dataset at each site; ER, total event rainfall (mm); Total Stormflow Q, total stormflow discharge during storm event determined via event separation (m^3^); Peak Q, event maximum flow recorded during storm event (m^3^ s^−1^); Lag peak to peak, the time between peak rainfall and peak discharge in a storm event (hours). For each metric the median value is presented along with the interquartile range and p value from Man–Whitney U tests (with statistical significance at the *p* < 0.05 level indicated by*). Q5:Q95 = flashiness index results showing ratio between high and low flow metric an increase in Q5:Q95 indicates increased flashiness and a reduction indicates a more attenuated flow regime.

Monitoring of these dam sequences as they mature will continue and may elucidate how hydrological response varies with magnitude or spatial configuration of beaver impact or how long it takes for stable and consistent flow attenuation to occur through a beaver impacted wetland.

#### 4.2.2 Flow attenuation during large events

For flood management there is a focus on the performance of different management approaches during large storms, where there is the greatest volume of water and therefore greatest flood risk. As identified by Westbrook et al. (2020) there has been a commonly held misconception that, due to their relatively small water storage capacity and potential to fail, beaver dams will cause limited attenuation during large rainstorms. To address this question Westbrook et al. (2020) monitored a large flood (200–350 mm over 4 days), concluding that beaver dam sequences can provide attenuation even in large storms. The authors attribute this to the persistence of the majority of dams and the transient storage of flood water in ponds. Data analysed herein did not include events of the magnitude of that recorded by Westbrook et al. (2020), with the largest rainfall event recorded in the 3+ years of post‐beaver monitoring being a 50.5 mm event at Budleigh Brook. However, continuous monitoring at sites resulted in >400 events for the Q5 event subset, demonstrating that the flow attenuation impact persisted for larger events. Due to their leaky nature, water storage in beaver dams is temporally variable (Karran et al., 2016; Puttock et al., 2017) and therefore capacity for attenuation is transient rather than finite during and between storm events.

At the more established sites (Woodland Valley and Budleigh Brook), reductions in peak flow increased during larger events. This supports Nyssen et al. (2011) who showed that, in a mountainous stream in Belgium with a sequence of six dams, peak flow attenuation for the highest discharges was greater than for smaller events. Therefore, in agreement with Butler and Malanson (2005) and Puttock et al. (2017) it is proposed that increased water storage and the slowed release of water through dams, can deliver flow attenuation for large storm events across multiple scales.

#### 4.2.3 Seasonal variation

A somewhat unexpected finding from this study was that, not only did flow attenuation persist and at two sites increase during large flood events, but at the same sites (Woodland Valley and Budleigh Brook) greater flood flow attenuation was observed during the wet season. Water levels in beaver ponds vary significantly as a result of meteorological conditions (Puttock et al., 2017; Westbrook et al., 2020), particularly in areas with large seasonal variations in flow, for example, due to snowmelt (Majerova et al., 2015) or ephemeral drylands. However, given the consensus that flow attenuation is primarily due to water storage, greater attenuation during wet periods is surprising in a temperate climate. It might be expected that, in the wet season, an increase in the magnitude and frequency of rainfall events, combined with reduced vegetation cover, reduced evapotranspiration losses and an increase in saturated soils and runoff would result in the opposite effect. A possible explanation is that, during drier periods; (i) as observed by Nyssen et al., 2011 and others, beaver activity results in increased flows and (ii) the overall smaller storm events typically experienced during the dry seasons can flow through the leaky dams (conceptualized by Neumayer et al. (2020) as a series of pipes through a barrier by which water can flow), whilst the more intense storm flows experienced during winter, back up against dams, which maintain enough “leakiness” and consequent “freeboard” to ensure storage is transient enough to provide ongoing attenuation capacity, but enough of a barrier to significantly reduce the flood peak flows experienced during wet seasons.

It must be acknowledged that this seasonal variation was not observed at the other less mature sites (Forest of Dean and Yorkshire). Although, whether this inconsistency was due to forest landscapes showing less seasonal variation or whether it was because these two sites were younger and less beaver impacted is not clear. What is clear is that a greater understanding of the mechanisms by which beaver dam sequences and associated wetlands alter flow regimes through a range of flow and seasonal conditions is still required.

### 4.3 Implications for catchment and natural flood management

Recent years have seen a growing interest in natural catchment management strategies (Dadson et al., 2017). For instance, in England, “Working With Natural Processes” (WWNP) and Natural Flood Management (NFM) is now incorporated into government policy (Environment Agency, 2017). It has been suggested that wetland re‐creation, woody debris dams and floodplain reconnection, can all play a significant role in reducing downstream flooding (Lane et al., 2004; Ockenden et al., 2012; Pettorelli et al., 2018.; Wharton & Gilvear, 2007; Wilkinson et al., 2010).

There is growing understanding of where beavers can dam (Graham et al., 2020; Macfarlane et al., 2015) and how beavers will utilize catchments (Brazier, Elliott, et al., 2020; Brazier, Puttock, et al., 2020; Campbell‐Palmer et al., 2018; Halley et al., 2020). However, European catchments have become dominated by human activity (Brown et al., 2018; Hewett et al., 2020) and, as a truly nature based approach, it must be recognized and reconciled that managers will not have the level of control over beaver engineering they do over human engineering (as indeed, we as researchers did not). Beavers will bring unique but manageable issues (Campbell‐Palmer et al., 2016); stakeholder and public engagement will therefore be required to mitigate the risk of conflict (Auster et al., 2019).

NFM now covers a range of approaches from those that are engineered in line with precise flood risk mitigation specifications to those that are more akin to “rewilding” giving space to allow the reinstatement of natural processes (Lawton et al., 2010). Beavers may sit uncomfortably with approaches towards the engineered end of the spectrum, that is, the catchment systems engineering approach proposed by Hewett et al. (2020) which advocates a combination of hard engineering with catchment interventions that mimic natural processes. Within such approaches, the highly dynamic nature of beaver engineering may be deemed risky. In contrast, beaver engineering sits more comfortably within restoration approaches that advocate restoring natural structure and function to catchments including biomic river restoration or Stage 0 approaches (Cluer & Thorne, 2014; Johnson et al., 2019) or proposals to return our riverine ecosystems to pre‐Anthropocene dynamic equilibrium (Brown et al., 2018). Such approaches could embrace the dynamic nature of beaver, whilst conflicts could be minimized and a host of other ecosystem service benefits provided (Dalbeck et al., 2020; Law et al., 2016, 2017). Perhaps the most pragmatic way forward is an open‐minded holistic assessment on catchment scales to determine where more tightly constrained engineering approaches are required and where more natural multi‐benefit approaches could be encouraged.

This study supports the conclusion of Westbrook et al. (2020) (albeit from research in a very different Canadian landscape) that, while beaver dam sequences are unlikely to provide 100% downstream flood protection, they can transiently store water and attenuate flood flows. It is thus argued, that the results provided herein, and research they build upon, that is, (Law et al., 2016; Nyssen et al., 2011; Puttock et al., 2017), support the incorporation of beavers into multiple‐benefit catchment management strategies that embrace natural flood management objectives. However, to maximize the effectiveness of beavers a greater understanding of the density and distribution of beaver dams needed to mitigate downstream flooding effectively, is required. Further research, should incorporate both empirical studies to gain a greater mechanistic understanding of beaver dam sequences and wetlands, combined with development of modelling approaches to upscale robustly such understanding and facilitate adoption by the flood management community.

## 5 CONCLUSION

Results demonstrate that the dams and associated complex wetlands that beaver engineering creates, can alter flood flow regimes. Statistical analysis, across the multiple sites in England presented herein adds significant confidence to the assertion that beaver engineered landscapes can result in significant flood flow attenuation following rain storm events. Critically, results quantitatively demonstrate that the peak flow reductions, observed after beaver dam complex construction, persist during both the wet times of the year and during large events when the societal, economic and environmental risks of flooding are greatest.

Results also showed that, across all sites, the overall “flashiness” of flow regimes was reduced. This suggests that the increased water storage resulting from the creation of beaver ponds and wetlands could also play a base flow maintenance role during dry, low flow periods, creating a valuable ecological refuge and potentially increasing the sustainability of water supplies. The hydrological behaviour of beaver‐impacted systems during drought periods is a promising avenue for further research to quantify whether beaver engineering has significant benefits during both hydrological extremes, that is, floods and droughts.

The exact impact of beaver will be site specific to an extent, depending on the level of engineering and the structure of the ecosystem. Further research should aim to contribute greater mechanistic understanding of how dams and dam sequences drive the flow attenuation impact observed herein. Results demonstrated the strength of BACI analysis for empirical hydrological analysis and we advocate the wider use of this analysis in related studies. A mechanistic understanding of beaver systems across different environments and climatic zones would also be beneficial. Combined with modelling approaches, this increase in empirical understanding could enable prediction of the catchment outlet effects of cumulative dam complexes across a range of beaver impact scenarios, up to the impact from a widespread return of beaver to all headwater streams. Alongside the well documented biodiversity benefits of beaver, results presented demonstrate that beaver could, with appropriate management, provide a valuable component of more natural catchment management approaches, increasing the resilience of landscapes to extreme climatic events.

## Supporting information


**Data S1.** Links to full data repositories and code for data analysis undertaken.

**Figure S1.** An example event extraction time series output for Woodland Valley. Full size time series plots for Woodland Valley, Forest of Dean, Yorkshire, Budleigh Brook and Colaton Brook – Control are included in the data repository: https://github.com/exeter-creww/Combined_Beaver_Hydro/tree/main/2_Event_Extraction/OutputPlots_TIMESERIES.

**Figure S2.** Boxplot showing peak flow results across all sites: EBUD, Budleigh Brook; FoD, Forest of Dean; WV, Woodland Valley; Yorkshire, Yorkshire.

**Figure S3.** Boxplot showing total event rainfall results across all sites: EBUD, Budleigh Brook; FoD, Forest of Dean; WV, Woodland Valley; Yorkshire, Yorkshire.

**Figure S4.** GLM model results between peak Q and total event rainfall for a Q5 large event dataset, before and after beaver impact at Budleigh Brook and compared to a control site (Colaton Brook). Top: model output plots; Bottom: model summary and marginal mean values for each site.

**Figure S5.** Multi‐panel figure showing aerial view of each site for context. Top left = Woodland Valley, top right = Budleigh Brook, bottom left = Yorkshire, bottom right = Forest of Dean.

**Figure S6.** Example of dams at site showing difference between where a dam stretches across a more developed site connecting the channel and floodplain (Left) in this case at Woodland Valley and a less developed site where dam activity at the time of monitoring was restricted to in‐channel (Right) at the Forest of Dean.

## Data Availability

The data that support the findings of this study are openly available in Github at https://github.com/exeter-creww. Full details of repositories for each section of data analysis are provided in S.I.1. This is publically available under a GNU General Public Licence (GPL‐3.0 License).
