# Supplementary material for: Beaver dams attenuate flow: A multi‐site study
Source: Hydrol Process. 2021 Jan 1;35(2):e14017. doi: 10.1002/hyp.14017 (PMC7898794; doi:10.1002/hyp.14017)
Supplement: Supplementary file 1 — Data S1. Links to full data repositories and code for data analysis undertaken. [file HYP-35-na-s001.docx]

Supplementary Information 1.(SI.1)

Beaver dams attenuate floods: a multi-site, multi-scale study

Authors: Alan Puttock*, Hugh A. Graham, Josie Ashe, David J. Luscombe and Richard E. Brazier

*lead and corresponding author. Email: a.k.puttock@exeter.ac.uk

Data analysis

Links below provide access to public repositories for data analysis undertaken:

https://github.com/exeter-creww/Rainfall_radar

For Code relating to the extration of MetOffice NIMROD rainfall radar data

https://github.com/exeter-creww/Combined_Beaver_Hydro

Data undertaken for event extraction and before and after hydrological impacts of beaver as detailed in Puttock et al (In Prep) Beaver dams attenuate floods: a multi-site, multi-scale study

https://github.com/exeter-creww/Budleigh_Brook_Beaver_Hydro

Data undertaken for event extraction and Before After Control Impact (BACI) analysis for Budleigh Brook as part of Puttock, et al. (in prep).
