# Supplementary figures and images for: Beaver dams attenuate flow: A multi‐site study
Source: Hydrol Process. 2021 Jan 1;35(2):e14017. doi: 10.1002/hyp.14017 (PMC7898794; doi:10.1002/hyp.14017)

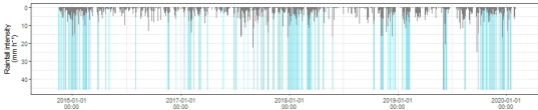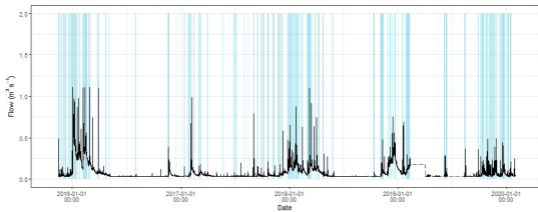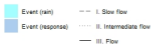

Supplement: Supplementary file 2 — Figure S1. An example event extraction time series output for Woodland Valley. Full size time series plots for Woodland Valley, Forest of Dean, Yorkshire, Budleigh Brook and Colaton Brook – Control are included in the data repository: https://github.com/exeter-creww/Combined_Beaver_Hydro/tree/main/2_Event_Extraction/OutputPlots_TIMESERIES. [file HYP-35-na-s002.pdf]

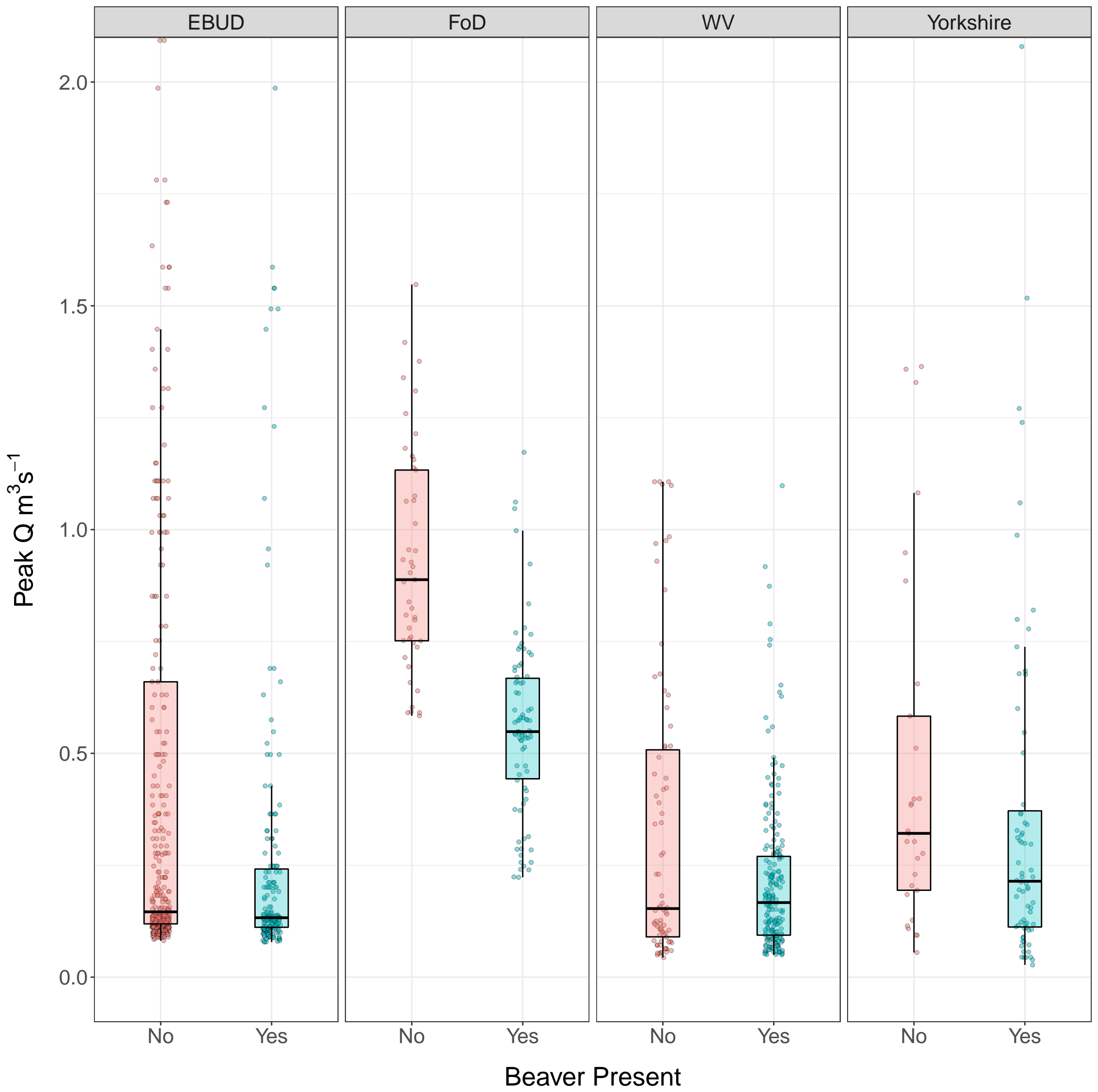

Supplement: Supplementary file 3 — Figure S2. Boxplot showing peak flow results across all sites: EBUD, Budleigh Brook; FoD, Forest of Dean; WV, Woodland Valley; Yorkshire, Yorkshire. [file HYP-35-na-s003.pdf]

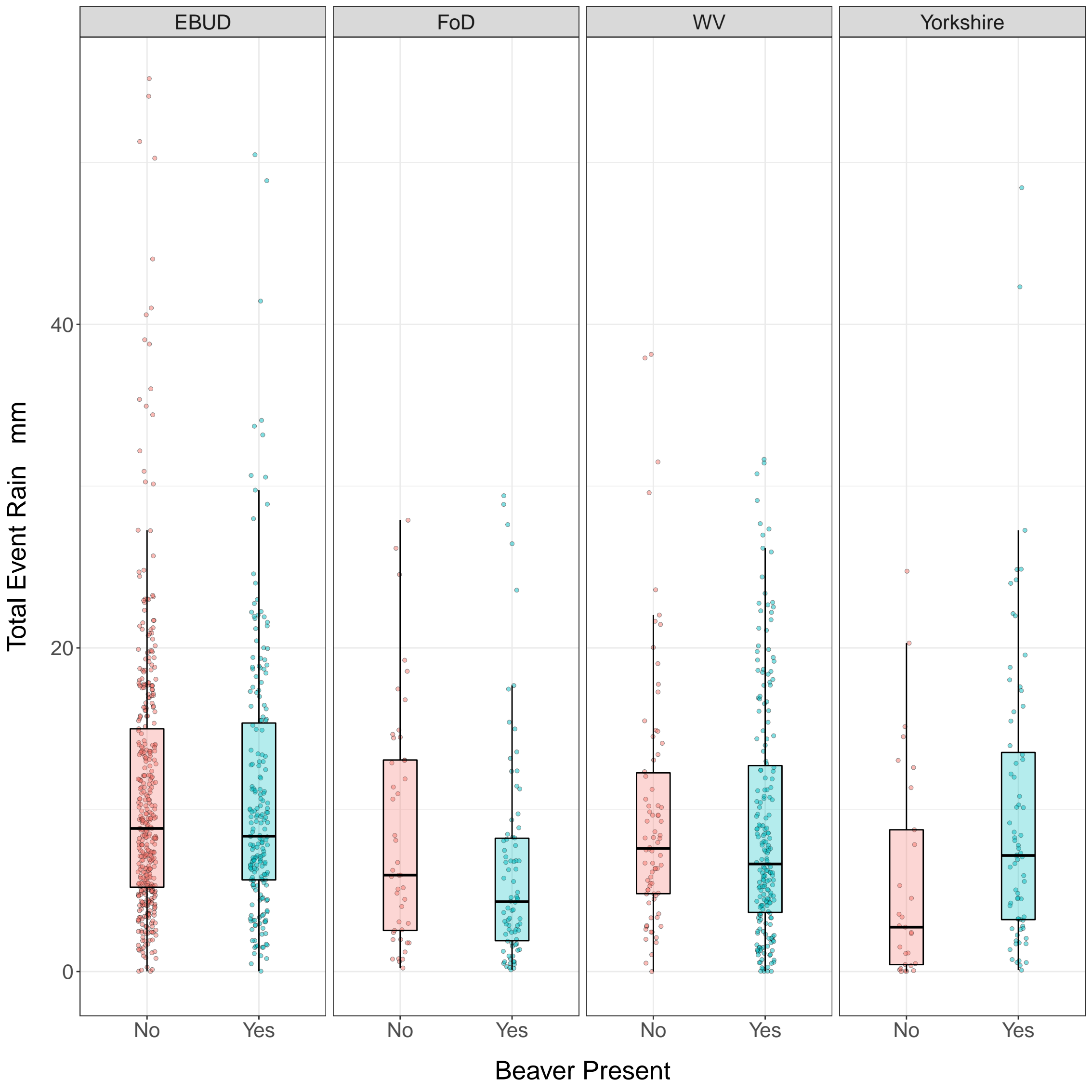

Supplement: Supplementary file 4 — Figure S3. Boxplot showing total event rainfall results across all sites: EBUD, Budleigh Brook; FoD, Forest of Dean; WV, Woodland Valley; Yorkshire, Yorkshire. [file HYP-35-na-s004.pdf]

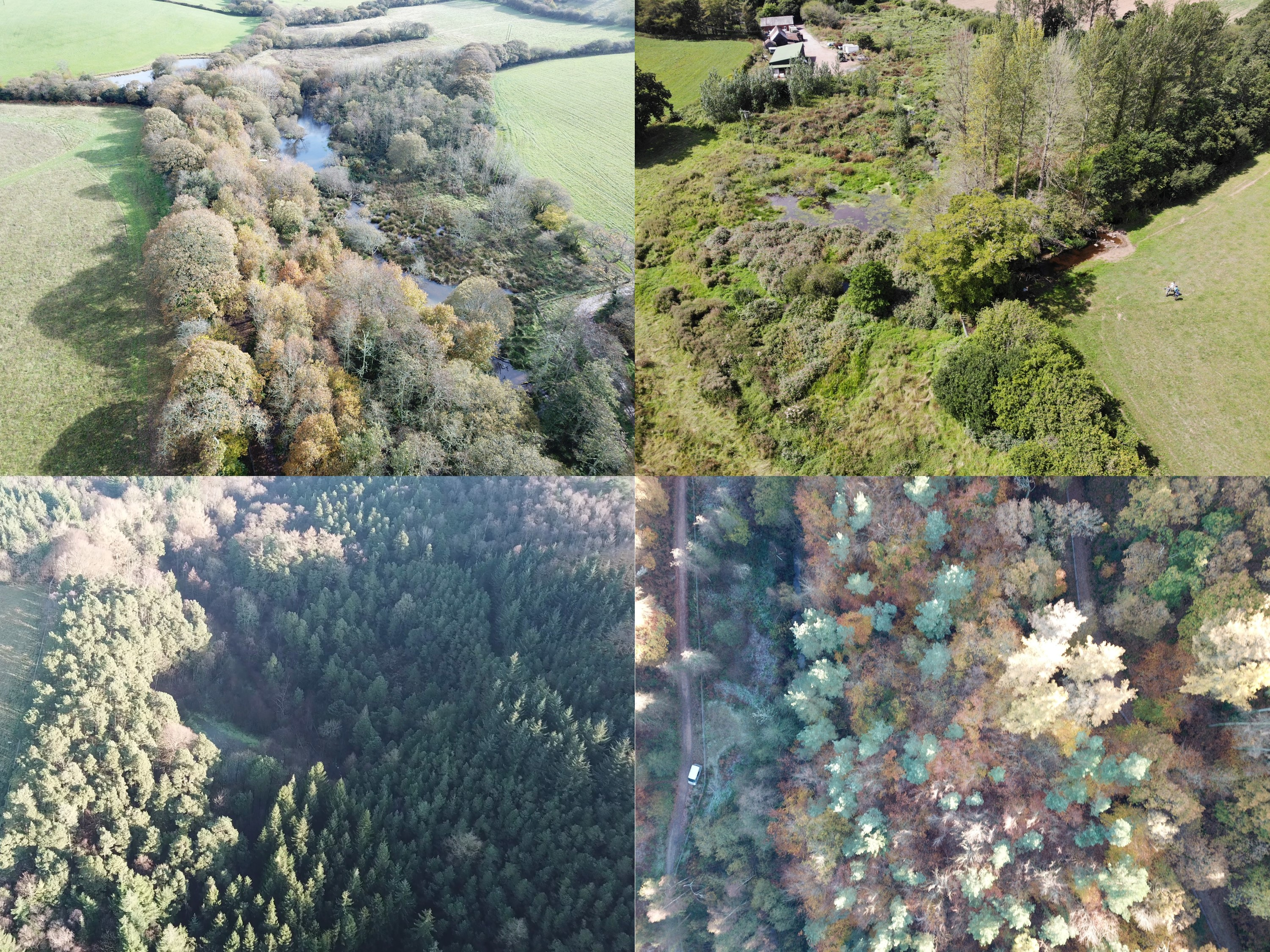

Supplement: Supplementary file 6 — Figure S5. Multi‐panel figure showing aerial view of each site for context. Top left = Woodland Valley, top right = Budleigh Brook, bottom left = Yorkshire, bottom right = Forest of Dean. [file HYP-35-na-s006.tif]

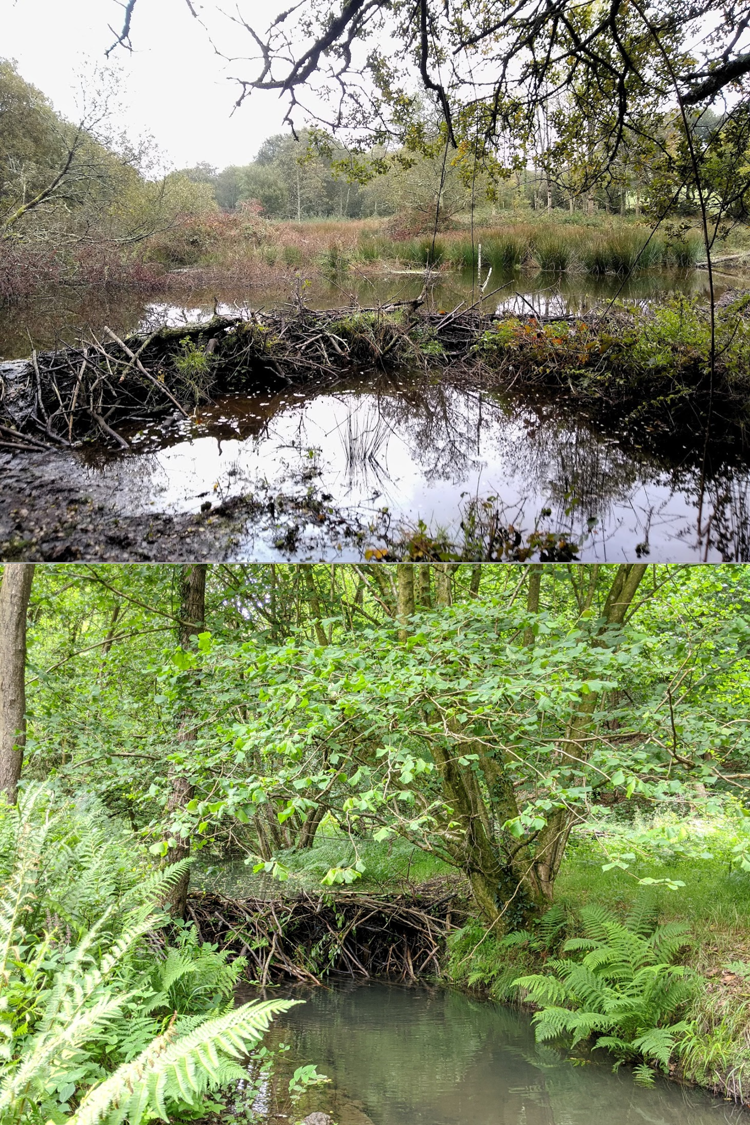

Supplement: Supplementary file 7 — Figure S6. Example of dams at site showing difference between where a dam stretches across a more developed site connecting the channel and floodplain (Left) in this case at Woodland Valley and a less developed site where dam activity at the time of monitoring was restricted to in‐channel (Right) at the Forest of Dean. [file HYP-35-na-s007.tif]
