# Supplementary material for: Beaver dams attenuate flow: A multi‐site study
Source: Hydrol Process. 2021 Jan 1;35(2):e14017. doi: 10.1002/hyp.14017 (PMC7898794; doi:10.1002/hyp.14017)
Supplement: Supplementary file 5 — Figure S4. GLM model results between peak Q and total event rainfall for a Q5 large event dataset, before and after beaver impact at Budleigh Brook and compared to a control site (Colaton Brook). Top: model output plots; Bottom: model summary and marginal mean values for each site. [file HYP-35-na-s005.pdf]

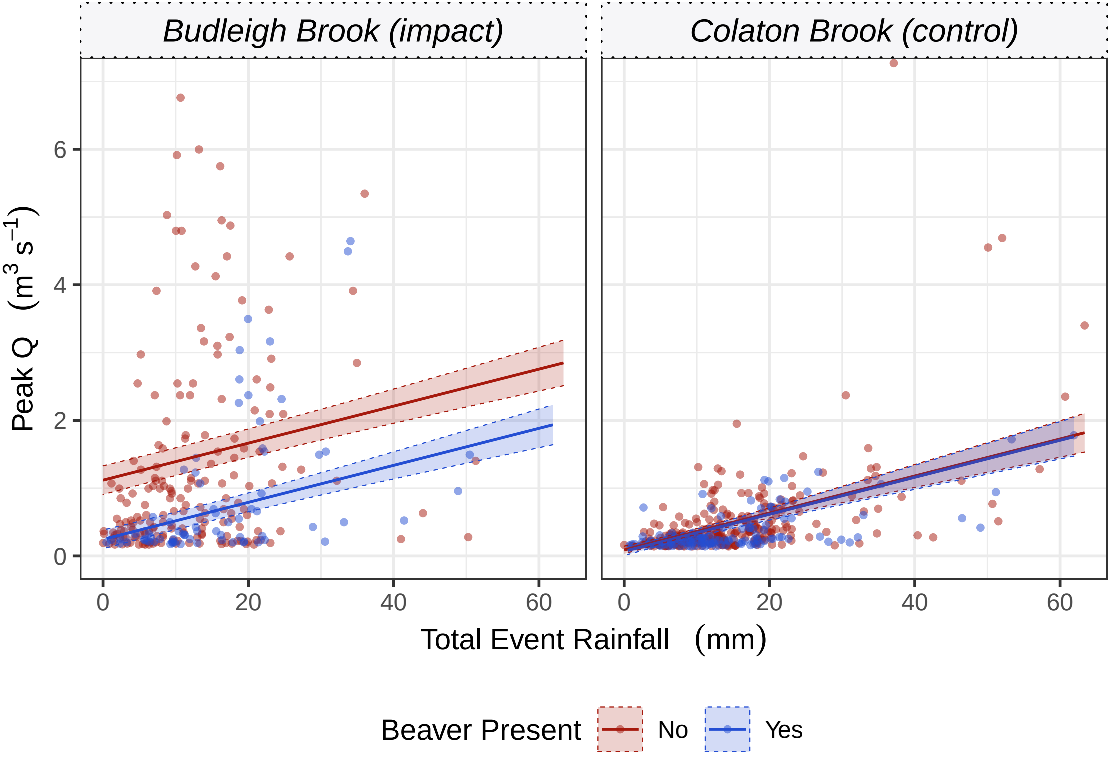

Budleigh:Colaton Q5 Regression Summary

term	estimate	std.error	T.statistic	p.value
Intercept	0.088	0.029	3.058	0.002 *
Total Rainfall	0.027	0.003	10.570	< 0.001 **
Beaver	-0.020	0.034	-0.578	0.564
Budleigh Brook	1.030	0.107	9.662	< 0.001 **
Beaver:Budleigh Brook	-0.854	0.128	-6.676	< 0.001 **

Marginal Means

Beaver	Site	estimate	std.error
No	Colaton Brook (control)	0.497	0.028
Yes	Colaton Brook (control)	0.477	0.034
No	Budleigh Brook (impact)	1.527	0.105
Yes	Budleigh Brook (impact)	0.653	0.067
